# Supplementary material for: GrandPrix: scaling up the Bayesian GPLVM for single-cell data
Source: Bioinformatics. 2018 Jul 2;35(1):47–54. doi: 10.1093/bioinformatics/bty533 (PMC6298059; doi:10.1093/bioinformatics/bty533)
Supplement: Supplementary Data [file bty533_supplementary_data.pdf]

Supplementary material for 'GrandPrix: Scaling up the Bayesian GPLVM for single-cell data'.

Sumon Ahmed[1], Magnus Rattray[1] and Alexis Boukouvalas[1]

[1]Division of Informatics, Imaging and Data Sciences Faculty of Biology, Medicine and Health
University of Manchester
May 1, 2018

Contents

1 Derivation of model **1**
1.1 Sparse GP Regression 2
1.2 Sparse GPLVM 4
1.3 Pseudotime estimation using the GPLVM 6

2 Comparison with the DeLorean model **7**
2.1 Inferring withheld time points and smooth pseudotime trajectories 7
2.2 Correctly identifying precocious cells 9
2.3 Recovering cell cycle peak times 9

3 Recovering Diffusion Pseudotime (DPT) **10**

4 2-D visualization of ∼68k Peripheral Blood Mononuclear Cells (PBMCs) **10**

5 Identifying branching dynamics in mouse embryonic stem cells **13**

1 Derivation of model

Sparse approximations are useful techniques for practical inference of Gaussian processes (GP) to deal with large datasets. Sparse approximations rely on a small number of parameters termed inducing or auxiliary points that approximate the posterior distribution over functions. The inducing points may be chosen from the training dataset or be an arbitrary set such as a uniform grid in the input space. Further they may be treated as constant or be optimised with respect to the model likelihood. The two most popular inducing point approximations for GPs are the Fully Independent Training Conditional (FITC) (Snelson and Ghahramani, 2006) and the Variational Free Energy approximation (VFE) (Titsias, 2009). The two methods differ in both theoretical aspects as well as how the inducing points are handled.

In this work, we have used the VFE approximation that tries to maximise a lower bound to the exact marginal likelihood in order to select the inducing points and model hyperparameters jointly. It minimises the KL divergence between the variational GP and the full posterior GP which allows it to avoid overfitting as well as to approximate the exact GP posterior. In Subsection 1.1, we describe briefly the derivation of a variational lower bound for sparse GP regression. We discuss the properties of the VFE approximation and highlight how it differs theoretically from the FITC approach used in the DeLorean approach of Reid and Wernisch (2016). In Subsection 1.2, we discuss the derivation of an exact lower bound for the sparse Bayesian Gaussian Process Latent Variable Model (GPLVM). We highlight some issues that may emerge in practical applications and how to address them.

1.1 Sparse GP Regression

In GP regression, our interest is to model the latent function $f(.)$, given a training dataset $\{X, Y\}$ of N inputs $\{\mathbf{x}_1, ..., \mathbf{x}_N\}$ and their corresponding noisy observations $\{\mathbf{y}_1, ..., \mathbf{y}_N\}$. Let the dimensionality of inputs and observations be Q and D respectively and $F = \{\mathbf{f}_1, ..., \mathbf{f}_N\}$ the collection of latent noise-free outputs.

$$X \in \mathbb{R}^{N\times Q}$$
$$F \in \mathbb{R}^{N\times D}$$
$$Y \in \mathbb{R}^{N\times D}$$

In GP regression , we define a Gaussian Process prior over the latent function space, where each output is modelled by a separate GP. If GPs are taken to be independent across the dimensions then we have the prior distribution as follows

$$p(F|X) = \prod_{d=1}^{D} p(\mathbf{f}_d|X), \tag{1}$$

where $\mathbf{f}_d$ is the dth column of the matrix F and

$$p(\mathbf{f}_d|X) = \mathcal{N}(\mathbf{f}_d|\mathbf{0}, K_{NN}). \tag{2}$$

Here $K_{NN} = k(X, X)$ is the covariance matrix of size $N \times N$ and is defined by the covariance function k. Data points Y are the noisy observations and possess a one-to-one relation with the latent function values F. Considering independent and identically distributed (i.i.d.) Gaussian noise with precision β, we have

$$p(Y|F) = \prod_{d=1}^{D} p(\mathbf{y}_d|\mathbf{f}_d, \beta), \tag{3}$$

where

$$p(\mathbf{y}_d|\mathbf{f}_d, \beta) = \mathcal{N}\left(\mathbf{y}_d|\mathbf{f}_d, \beta^{-1}I_N\right). \tag{4}$$

In regression, X is given and we can analytically integrate out F to get the marginal likelihood function

$$p(Y|X) = \left(\int p(Y|F)p(F|X)dF\right) = \prod_{d=1}^{D} \mathcal{N}\left(\mathbf{y}_d|\mathbf{0}, K_{NN} + \beta^{-1}I_N\right). \tag{5}$$

Evaluating the likelihood function of Equation (5) requires the inversion of the $N \times N$ covariance matrix K_{NN} which needs $\mathcal{O}(N^3)$ time complexity, thus making it impractical for large datasets. To reduce the computational complexity, sparse approximations use a collection of M inducing points instead of the collection of all training input points which reduces the computational complexity to $\mathcal{O}\left(NM^2\right)$ with $M << N$. The inducing points are defined to be in the same space as the inputs X. The latter may contain a lot of redundancy as only a few points may be needed to describe domains where the response is smoothly changing; the inducing point formalism allows to place the majority of the points in areas where the function response is complex, such as around discontinuities and changepoints. The collection of inducing points aim to summarise the characteristics of the latent function space and are treated as sufficient statistics for the entirety of training data. Let U the latent function values at the M inducing points Z

$$U \in \mathbb{R}^{M\times D}$$
$$Z \in \mathbb{R}^{M\times Q}$$

A Bayesian treatment of the sparse GP regression model requires the analytical computation of the log marginal likelihood. After introducing the inducing points, the log marginal likelihood of the model takes the following from

$$\log p(Y|X) = \log \int p(Y|F)p(F|X,U)p(U|Z)dUdF, \tag{6}$$

where

$$p(U|Z) = \prod_{d=1}^{D} p(\mathbf{u}_d|Z). \tag{7}$$

Here $\mathbf{u}_d$ is the dth column of the matrix U and

$$p(\mathbf{u}_d|Z) = \mathcal{N}(\mathbf{u}_d|\mathbf{0}, K_{MM}) \tag{8}$$

is the marginal GP prior over the inducing points. K_{MM} is the covariance matrix constructed over the M inducing inputs Z. Titsias (2009) has considered the inducing points as variational parameters[1] which allows him to marginalise out (F,U) analytically for any values of Z. Taking this interpretation of the inducing points into consideration, we can drop out the notation Z to simplify our expressions. To remove the terms having K_{NN}^{-1}, the approximate variational distribution over the random variables have been factorised as follows

$$q(F,U) = p(F|X,U)q(U), \tag{9}$$

where $q(U)$ is a free-form distribution with

$$q(U) = \prod_{d=1}^{D} q(\mathbf{u}_d). \tag{10}$$

As U is the sufficient statistics of F, the optimisation of the variational distribution over Z tries to generate U such a way that makes F approximately conditionally independent from the observations Y given U. Now by using Jensen's inequality substituting Equation (9) into Equation (6)

$$\begin{aligned}\log p(Y|X) &= \log \int q(F,U)\frac{p(Y|F)\cancel{p(F|X,U)}p(U)}{\cancel{p(F|X,U)}q(U)}dUdF \\ &\geq \int q(U)\left(p(F|X,U)\log p(Y|F)dF + \log\frac{p(U)}{q(U)}\right)dU \\ &= \sum_{d=1}^{D}\int q(\mathbf{u}_d)\left(p(\mathbf{f}_d|X,U)\log p(\mathbf{y}_d|\mathbf{f}_d)dF + \log\frac{p(\mathbf{u}_d)}{q(\mathbf{u}_d)}\right)dU.\end{aligned} \tag{11}$$

The optimal form of the approximate distribution $q(\mathbf{u}_d)$ can be found by variational calculus and re-inserting it back to the Equation (11)(reversing Jensen's inequity) gives the lower bound of the marginal likelihood (Titsias, 2009)

$$\mathcal{F} = \sum_{d=1}^{D}\mathcal{F}_d, \tag{12}$$

where

$$\begin{aligned}\mathcal{F}_d &= \log\left[\mathcal{N}\left(\mathbf{y}_d|\mathbf{0}, Q_{NN} + \beta^{-1}I_N\right)\right] - \frac{\beta}{2}tr(T) \\ &= -\frac{N}{2}\log(2\pi) - \underbrace{\frac{1}{2}\log|Q_{NN}+G|}_{\text{complexity penalty}} - \underbrace{\frac{1}{2}\mathbf{y}_d^{\mathrm{T}}\left(Q_{NN}+G\right)^{-1}\mathbf{y}_d}_{\text{data fit}} - \underbrace{\frac{\beta}{2}tr(T)}_{\text{trace term}},\end{aligned} \tag{13}$$

[1]Variational parameters are neither hyperparameters nor random variables. They are an extra set of parameters that are used to minimise the distance of the approximating variational distribution to the full posterior GP density.

with

$$Q_{NN} = K_{NM}K_{MM}^{-1}K_{MN}, \qquad G_{\text{VFE}} = \beta^{-1}I_N, \qquad T_{\text{VFE}} = K_{NN} - Q_{NN}. \tag{14}$$

Here, Q_{NN} is approximating the true covariance matrix K_{NN}, but only involves the inversion of a $M \times M$ matrix K_{MM}. K_{MM} is the covariance matrix on the inducing inputs Z; K_{NM} is the cross covariance matrix between the training and inducing inputs, i.e. between X and Z and $K_{MN} = K_{NM}^{\text{T}}$. The objective function of Equation (12) consists of three terms. Bauer *et al.* (2016) have explained that the data fit term imposes a penalty on the data which fall outside of the covariance ellipse $(Q_{NN} + G)$. The complexity term represents the integral of the data fit term over all possible observations and characterises the volume of probable datasets which are compatible with the data fit term. Thus, the data fit and the complexity penalty can be considered directly analogous to the full GP. Finally, the trace term confirms that the objective function of Equation (12) is a true lower bound for VFE approximation. It penalises the total conditional variance of the prior $p(\mathbf{f}_d|U)$, conditioned on the inducing points. Thus the trace term highlights the fact that the VFE approximation not only models a specific dataset but also predicts the covariance structure K_{NN} of the full GP and without this term VFE may overestimate the marginal likelihood like previous methods of sparse approximation such as FITC. In fact, the objective function of the FITC can be obtained from Equation (12) by using the same expression for Q_{NN} and taking

$$G_{\text{FITC}} = \text{diag}\left[K_{NN} - Q_{NN}\right] + \beta^{-1}\mathbf{I}_N, \qquad T_{\text{FITC}} = 0, \tag{15}$$

which clearly shows that the objective function of the FITC can be obtained by modifying the GP prior

$$p(\mathbf{f}_d|U) = \mathcal{N}\left(\mathbf{f}_d|\mathbf{0}, Q_{NN} + \text{diag}\left[K_{NN} - Q_{NN}\right]\right). \tag{16}$$

Here the inducing points are acting as an extra set of hyperparameters to parametrise the covariance matrix Q_{NN}. As this approach changes the prior, the continuous optimisation of the latent variable $\mathbf{f}_d$ with respect to the inducing points U does not guarantee to approximate the full GP posterior (Titsias, 2009). Moreover, as $\mathbf{f}_d$ is heavily parametrised because of the extra hyperparameter U and the trace term is 0, overfitting may arise at the time of jointly estimating the inducing points and hyperparameters. To get a comprehensive study of FITC and VFE approximation methods in terms of both theoretical and practical aspects, see Bauer *et al.* (2016).

1.2 Sparse GPLVM

Gaussian Process Latent Variable Model can be considered as the unsupervised equivalent of GP regression. The model setup is same, but in the GPLVM case X is unobserved and needs to be treated as a random variable. Thus, the Bayesian treatment of GPLVM requires to marginalise out X. Titsias and Lawrence (2010) have developed a variational sparse Bayesian GPLVM using the same technique of variational sparse GP. From that point, the sparse GP can be thought as a special form of sparse GPLVM where the input variables X are given with zero variance.

Using the lower bound developed in the previous subsection, we can easily derive an exact lower bound for variational sparse Bayesian GPLVM. Let define the factorised prior distribution over the latent dimensions X

$$p(X) = \prod_{n=1}^{N} \mathcal{N}\left(\mathbf{x}_n|\boldsymbol{\mu}_n, \Lambda_n^{-1}\right) \tag{17}$$

where $\mathbf{x}_n$ is a row of the matrix X and Λ_n is the precision matrix. Now, by introducing a variational distribution $q(X)$ constrained to be Gaussian of the following form

$$q(X) = q(X|\mathcal{M}, \mathcal{L}^{-1}) = \prod_{n=1}^{N} \mathcal{N}\left(\mathbf{x}_n|\mathbf{m}_n, \mathcal{L}_n^{-1}\right) \tag{18}$$

with precision matrix $\mathcal{L}_n$ and by using the Jensen's inequality, we can write the log marginal likelihood as

$$
\begin{aligned}
\log p(Y) &= \log \int q(X) \frac{p(Y|X)p(X)}{q(X)} dX \\
&\geq \int q(X) \log \frac{p(Y|X)p(X)}{q(X)} dX \\
&= \int q(X) \log p(Y|X) dX - \int q(X) \log \frac{q(X)}{p(X)} dX \\
&= \mathbb{E}_{q(X)} \left[\log p(Y|X)\right] - \mathrm{KL}\left(q(X)||p(X)\right).
\end{aligned} \tag{19}
$$

Thus we have found a bound where the second term is the negative KL divergence between the distributions $q(X)$ and $p(X)$. As both of these distributions have been taken Gaussian, this term can be computed analytically. To compute the first term, we can use the bound developed in Equation (12). As Dai *et al.* (2014) show, the VFE GP and GPLVM methods are using the same variational bound with the formers expressions in X turned into expectations over $q(X)$. Let

$$
\tilde{\mathcal{F}} = \mathbb{E}_{q(X)} \left[\log p(Y|X)\right] = \sum_{d=1}^{D} \mathbb{E}_{q(X)} \left[\log p(\mathbf{y}_d|X)\right] = \sum_{d=1}^{D} \tilde{\mathcal{F}}_d. \tag{20}
$$

Now by using the lower bound of Equation (13)

$$
\begin{aligned}
\tilde{\mathcal{F}}_d = &-\frac{N}{2}\log(2\pi) - \frac{1}{2}\log\left|K_{MM}^{-1}\mathbb{E}_{q(X)}\left[K_{MN}K_{NM}\right] + \beta^{-1}\mathbf{I}_M\right| - \frac{1}{2}\mathbf{y}_d^{\mathrm{T}} W \mathbf{y}_d \\
&- \frac{\beta}{2} tr\left(\mathbb{E}_{q(X)}\left[K_{NN}\right] - K_{MM}^{-1}\mathbb{E}_{q(X)}\left[K_{MN}K_{NM}\right]\right),
\end{aligned} \tag{21}
$$

with

$$
W = \beta I_N - \beta^2 \mathbb{E}_{q(X)}\left[K_{NM}\right]\left(\beta\mathbb{E}_{q(X)}\left[K_{MN}K_{NM}\right] + K_{MM}\right)^{-1}\left(\mathbb{E}_{q(X)}\left[K_{NM}\right]\right)^{\mathrm{T}}.
$$

Let

$$
\psi_0 = tr\left(\mathbb{E}_{q(X)}\left[K_{NN}\right]\right),
$$

$$
\Psi_1 = \mathbb{E}_{q(X)}\left[K_{NM}\right],
$$

$$
\Psi_2 = \mathbb{E}_{q(X)}\left[K_{MN}K_{NM}\right].
$$

To calculate the exact bound of variational inference, we need to compute the Ψ statistics. Titsias and Lawrence (2010) have provided the calculation in a decomposable way across the latent variables of different observations. Intuitively, this decomposition is helpful when a new data vector is added to the training set as it can speed up the computations. Now ψ_0 can be written as

$$
\psi_0 = \sum_{n=1}^{N} \psi_0^n
$$

where

$$
\psi_0^n = \int k\left(\mathbf{x}_n, \mathbf{x}_n\right) \mathcal{N}\left(\mathbf{x}_n | \mathbf{m}_n, \mathcal{L}_n^{-1}\right) d\mathbf{x}_n. \tag{22}
$$

Ψ_1 is a $N \times M$ matrix, where

$$
(\Psi_1)_{nm} = \int k\left(\mathbf{x}_n, \mathbf{z}_m\right) \mathcal{N}\left(\mathbf{x}_n | \mathbf{m}_n, \mathcal{L}_n^{-1}\right) d\mathbf{x}_n, \tag{23}
$$

with $\mathbf{z}_m$ representing the mth row of the matrix Z. Ψ_2 is a $M \times M$ matrix and can be written as

$$\Psi_2 = \sum_{n=1}^{N} \Psi_2^n$$

where

$$(\Psi_2^n)_{mm'} = \int k(\mathbf{x}_n, \mathbf{z}_m) k(\mathbf{z}_{m'}, \mathbf{x}_n) \mathcal{N}\left(\mathbf{x}_n | \mathbf{m}_n, \mathcal{L}_n^{-1}\right) d\mathbf{x}_n. \tag{24}$$

Thus the computations of the Ψ statistics involve convolutions of the kernel function k with Gaussian densities which are tractable for many standard kernel functions. Analytic expressions of the statistics (ψ_0, Ψ_1, Ψ_2) for the RBF and linear kernels can be found in Titsias and Lawrence (2010); Damianou *et al.* (2016).

Now using the factorization adopted in the distributions $p(X)$ and $q(X)$, the KL divergence between them can be expressed as

$$\mathrm{KL}\left(q(X)||p(X)\right) = \frac{1}{2}\sum_{n=1}^{N}\left[\log\frac{|\mathcal{L}_n|}{|\Lambda_n|} + tr\left\{(\boldsymbol{\mu}_n - \mathbf{m}_n)\Lambda_n(\boldsymbol{\mu}_n - \mathbf{m}_n)^{\mathrm{T}} + \Lambda_n\mathcal{L}_n^{-1}\right\}\right] - \frac{nq}{2} \tag{25}$$

The close form exact variational lower bound of the Bayesian GPLVM can be found by putting the Equations (20) and (25) into Equation (19). This bound can be jointly maximised with model hyperparameters and variational parameters $\{\mathcal{M}, \mathcal{L}, Z\}$ by applying gradient based optimisation approaches. This is similar to the MAP optimisation approach adopted in the standard GPLVM (Lawrence, 2005), but here instead of optimising the random variables X, a set of variational parameters is optimised to get the full posterior distribution of X. Moreover, the set of inducing points Z are also considered as variational parameters, thus optimising over the inducing points further improves the approximation of the posteriors. The full derivation is available in Damianou *et al.* (2016).

However, the maximisation of the objective function/lower bound may be hindered by local optima as the likelihood surface is non-convex. To alleviate this issue, careful initialisations of both hyperparameters and variational parameters have to be made. In particular, the mean of the variational distribution $\mathcal{M}$ can be initialised based on the association with nearest neighbours of $\mathbf{y}_n$ in the observed data Y (Titsias and Lawrence, 2010). An alternative initialisation for $\mathcal{M}$ is to apply dimensional reduction techniques such as PCA, ICA, MDS, t-SNE etc. on the observed data and use the lower dimensional representation. In Zwiessele and Lawrence (2016), multiple dimension reduction methods are applied and using automatic relevance determination, the dominant two latent dimensions are used for visualisation.

It is well known that variational inference can underestimate the variance of the posterior distribution in some classes of model. The objective function of variational inference tries to minimise the KL divergence between the distributions $q(.)$ and $p(.)$ with respect to $q(.)$. As the KL divergence represents an expectation under the distribution $q(.)$, the objective function penalises placing mass in $q(.)$ on regions of latent dimensions where $p(.)$ is low, but penalises less where $q(.)$ has very little mass (Blei *et al.*, 2017). In other words, variational inference tries to fit a low variance estimate to the posterior density. However, Blei *et al.* (2006); Braun and McAuliffe (2010); Kucukelbir *et al.* (2017) have shown that variational approximation techniques do not necessarily suffer in terms of accuracy in predicting the posterior mean, which we have also found in our experimental results (see Section 2.1). Thus, by considering the speed-up benefits, variational inference is suggested as a suitable choice to handle larger datasets. A comprehensive study on the variational inference as well as how it differs from other optimisation techniques such as data subsampling can be found in Blei *et al.* (2017).

1.3 Pseudotime estimation using the GPLVM

The primary latent variables in our model are the one-dimensional pseudotimes t. Our model incorporates the capture time information of each cell in the form of an informative prior, and each pseudotime point has been drawn from a normal distribution

$$p(t) = \prod_{n=1}^{N} p(t_n) = \prod_{n=1}^{N} \mathcal{N}\left(t_n | \tau_n, \sigma_t^2\right) \tag{26}$$

where τ_n is the capture time of cell n and σ_t^2 is the prior variance over the pseudotime trajectory. The capture time prior variance σ_t^2 should be chosen carefully. It should be large enough so that the cells centred at capture time points τ_n are well spread through the entire time course. Choosing a very small value for σ_t^2 will narrow down the search space of pseudotime orderings and will restrict the inference process from discovering interesting regions. For a better results we suggest that the latent space should be scaled to the interval $[0, 1]$ and the prior variance should be chosen from the interval $[0.1, 1]$. We have also found that a smaller capture time prior variance works better when the data has more distinct capture time points and vice versa. Thus while working with the Guo *et al.* (2010) data set which has 7 distinct capture time points, we have found that a smaller $\sigma_t^2 = 0.1$ gives better results (see main paper, subsection 3.3).

The expression profile of each gene y_g is modelled from a Gaussian Process as a non-linear transformation of pseudotime, which is corrupted by some independent and identically distributed Gaussian noise with variance σ_{noise}^2

$$p(Y) = \prod_{g=1}^{D} y_g \sim \prod_{g=1}^{D} \mathcal{GP}\left(\mathbf{0}, \sigma^2 k(t, t^*) + \sigma_{noise}^2 \mathbf{I}\right), \tag{27}$$

where σ^2 is the process variance and $k(t, t^*)$ is the covariance function between two distinct pseudotime points t and t^*. The covariance function plays a fundamental role in GP methods and imposes a smoothness constraint shared by all genes. The automatic statistician (Duvenaud *et al.*, 2013) performs an automatic search over the space of covariance functions and has some heuristics to pick the best one. We have used the i.i.d.Gaussian noise with uniform variance across the whole pseudotime trajectory as in standard GPLVM (Titsias and Lawrence, 2010). However, a non-uniform noise model may be a more suitable choice for single-cell data as the variability of data points is a subject to change along the pseudotime trajectory. Moreover, a non-uniform noise model requires additional modelling of how the variability changes across pseudotime trajectory (Campbell and Yau, 2016). Misspecification of the latter may lead to misleading conclusions and we have therefore selected to use the simpler i.i.d.Gaussian noise with uniform variance in our approach. Similarly a count-based noise model may be a more appropriate choice for single cell data. However using a non-Gaussian noise model will make the model likelihood intractable and hence increase the computational complexity of inference. To learn more about sources of uncertainty in pseudotime inference, see Campbell and Yau (2016).

Further, we have extended the model to higher dimensional latent spaces x, which have been sampled from a standard normal distribution

$$p(x) = \prod_{n=1}^{N} p(x_n) = \prod_{n=1}^{N} \mathcal{N}\left(x_n | \mathbf{0}, \mathbf{I}\right). \tag{28}$$

Thus, we can express the latent spaces of our model as

$$X = \{\mathbf{x}_n\}_{n=1}^{N} = \{(t, x)_n\}_{n=1}^{N}. \tag{29}$$

Given the observed data Y (expression profiles), we can optimize our model based on the approach discussed above.

2 Comparison with the DeLorean model

We have applied our model on three different datasets from three different organisms which have been also used in Reid and Wernisch (2016). As we show in this section, the results produced by our model are similar to the DeLorean model, but our model converges significantly faster (see main paper).

2.1 Inferring withheld time points and smooth pseudotime trajectories

Windram *et al.* (2012) generated time series data from the effects of *Botrytis cinera* infection on *Arabidopsis thaliana*. The time series were measured over 48 hours for both infected and control conditions. The data contain 24 distinct capture time points where the expression level of each time point is measured in 2 hour intervals. These 24 times points have been grouped into 4 separate groups, each consisting of 6 consecutive time points, which have been fed to the model for prior initialisation. Thus the challenge was to infer the exact capture time for the samples when the actual capture times were withheld.

The inference process uses the cells in the infected condition. We have used the $\text{Matern}_{3/2}$ kernel (see main paper) and set the capture time prior variance to $\sigma_t^2 = 9$ as in Reid and Wernisch (2016). In Figure 1(a), we have plotted the estimated pseudotime using 10 inducing points against the actual capture time and it shows a close correspondence between them.

The means of the estimated pseudotime distribution from the DeLorean and GrandPrix models are in good agreement as evident by the high spearman correlation (0.996). Both models also possess similar posterior variance. The average standard deviation of the 24 time points is 0.53 in the GrandPrix model and 0.54 in the DeLorean ADVI model. The DeLorean MCMC model has an higher average sample standard deviation (0.98). Having unlimited computational resources, the sample variance of an MCMC algorithm is expected to approach the true distribution variance. It therefore seems likely that both variational algorithms are underestimating the posterior variance for this dataset. In scenarios where the posterior variance over pseudotime is important, such as when assessing the uncertainty of pseudotime assignments (Campbell and Yau, 2016), the potential underestimation of the variance by both variational approaches means that the MCMC approach would be preferable if it were computationally feasible. Unfortunately, the DeLorean MCMC approach is not computationally feasible when the number of cells is larger than a few hundred (Saelens *et al.*, 2018).

(a) With informative prior (b) Without prior

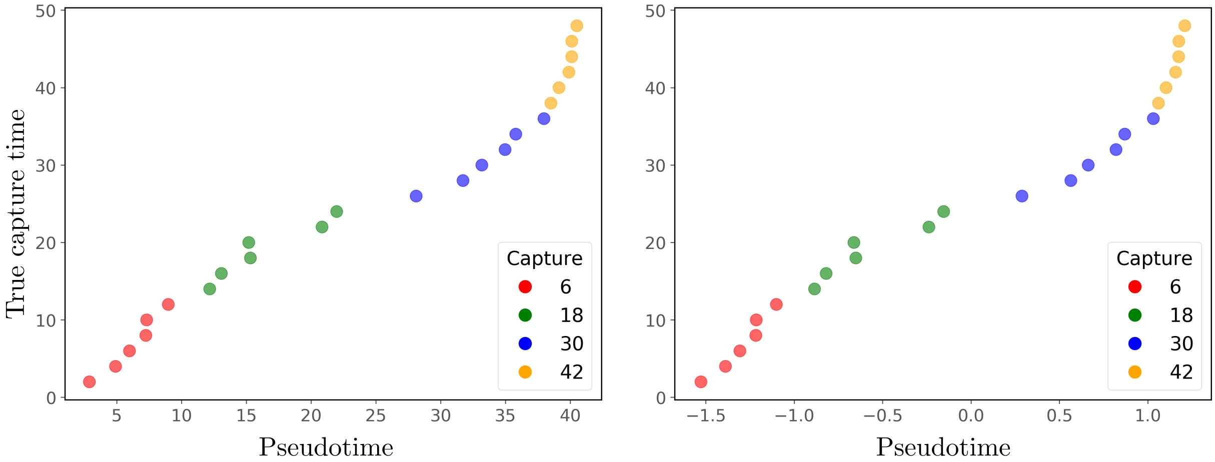

Figure 1: *Arabidopsis thaliana* microarray data (Windram *et al.*, 2012): Pseudotime posterior mean (horizontal axis) vs true capture time. Colours denote the prior information used by the GPLVM model. Our model successfully estimates pseudotimes that are in good agreement with the actual capture times.

To analyse the model's capability of estimating smooth pseudotime trajectory, we have calculated the roughness statistics R_g for varying number of inducing points. In each case, we have used 50 held out genes and 20 different initialisations. Table 1 shows the average R_g of our estimated pseudotimes which shows the model's potential to produce smooth trajectories even when using a small number of inducing points.

Although we have found that the incorporation of capture time in the form of prior information is helpful, GrandPrix can estimate the pseudotime ordering accurately without the presence of any prior information. Figure 1(b) shows the actual capture time against the GrandPrix estimated pseudotime ordering without using informative prior. It is evident that these two quantities are in good agreement

Table 1: Average roughness statistics of estimated pseudotime for *Arabidopsis thaliana* data.

# inducing points	4	6	8	10	12	14	16	18	20	22
Average Roughness R_g	0.737	0.713	0.709	0.706	0.706	0.706	0.706	0.706	0.706	0.706

and possess a high rank correlation (∼0.998).

2.2 Correctly identifying precocious cells

Shalek *et al.* (2014) identified a core antiviral gene module expressed in LPS after 2-4 hours. They found two cells captured at the 1 hour mark which switched to this group precociously. That is, these two cells have genes expressed which are not expressed in the other cells of the 1 hour group. It reflects the concept that some cells can progress faster through the differentiation process. Thus the challenge is to assign these two precocious cells at a later pseudotime than the other cells in the 1 hour group which will confirm the model's capability to uncover the developmental time line overruling the capture time prior information.

To fit the model, we have used 307 cells from the LPS time course which includes the two precocious cells. Among the several gene modules, the inference process used the top 74 most variably expressed genes from the clusters Id, IIIb, IIIc, IIId as in Reid and Wernisch (2016). Here we have used the Matern$_{3/2}$ kernel (see main paper) and set the capture time prior variance to $\sigma_t^2 = 1$.

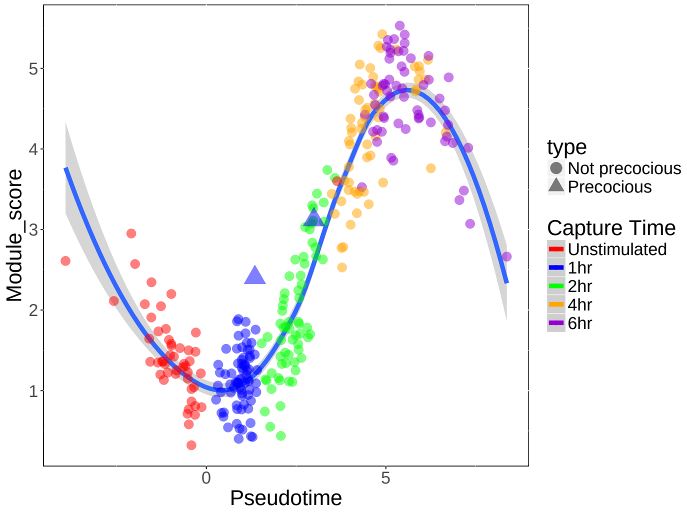

Figure 2: Mouse dendritic cells (Shalek *et al.*, 2014): The module score of core antiviral cells over pseudotime. The two precocious cells (plotted as triangles) have been placed in later pseudotimes than the other cells captured at 1 hour. A Loess curve (solid blue line) has been plotted thorough the data.

Fig. 2 shows the convergence of our model using 30 inducing points for the mouse dendritic cell data. The module score (Shalek *et al.*, 2014) of core antiviral genes have been plotted over the estimated pseudotime points. Two precocious cells have been assigned pseudotimes in the middle of the 2 hour group rather than in the 1 hour group. Thus, the model correctly infers that some cells can progress across the differentiation trajectory faster than others.

2.3 Recovering cell cycle peak times

McDavid *et al.* (2014) assayed the expression profiles of 333 genes in 930 cells by using single cell nCounter. In this work, we have used the expression data from the PC3 human prostate cancer cell line. The inference has been carried out by using the top 56 differentially expressed genes in 361 cells. The cells identified as G0/G1, S and G2/M by McDavid *et al.* (2014) have been mapped to

the capture times of 1, 2 and 3, respectively as in Reid and Wernisch (2016). Due to the additional challenge of optimizing pseudotime parameters for periodic data, we follow the approach of Reid and Wernisch (2016) where we have used as initial conditions random pseudotimes having the largest log likelihood to estimate cell cycle peak time points. We have used a periodic kernel (see main paper) and capture time prior variance $\sigma_t^2 = \frac{1}{4}$. This ensures the modelling of periodicity in the gene expression corresponding to the different cell cycle stages. The expression profiles of some selected genes over the estimated pseudotime (using 20 inducing points) are shown in Figure 3.

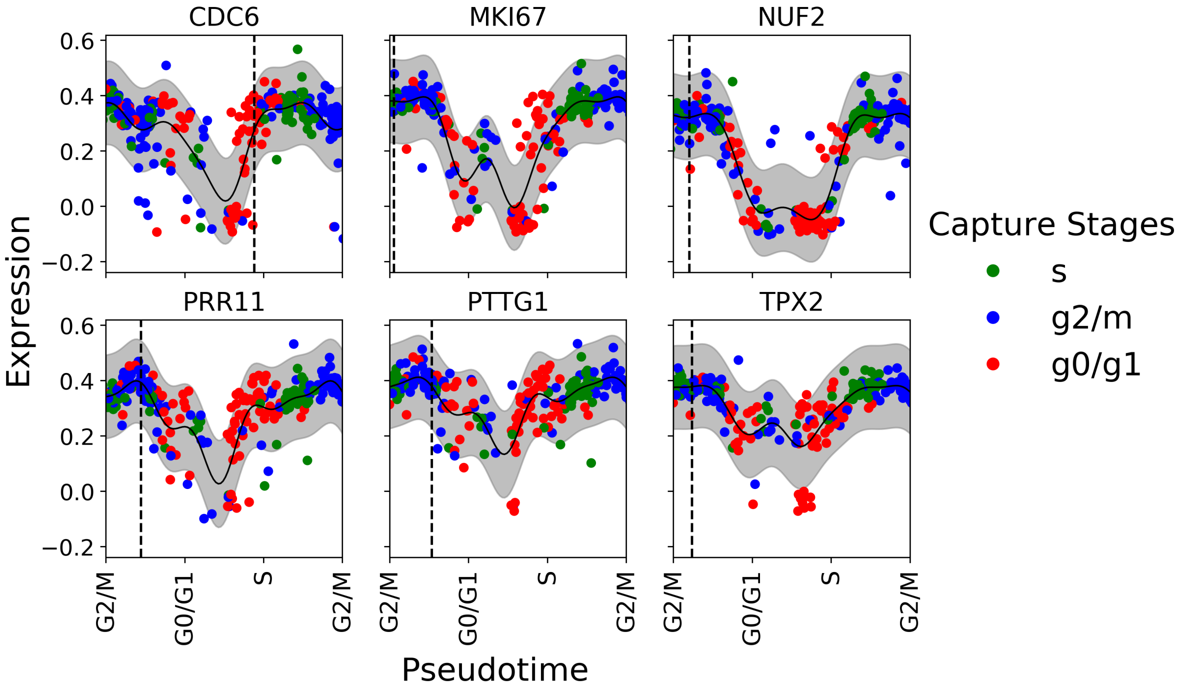

Figure 3: Cell cycle data of PC3 human prostate cancer cell line (McDavid *et al.*, 2014): Expression profiles over estimated pseudotime for some selected genes from PC3 human prostate cancer cell line. Each point corresponds to a particular gene expression in a cell. The points are coloured based on cell cycle stages according to McDavid *et al.* (2014). The circular horizontal axis (where both first and last labels are G2/M) represents the periodicity realized by the method in pseudotime inference. The solid black line is the posterior predicted mean of expression profiles while the grey ribbon depicts the 95% confidence interval. The vertical dotted lines are the CycleBase peak times for the selected genes.

In the CycleBase database (Santos *et al.*, 2014), the cell cycle related genes are arranged according to their peak times. To evaluate the models performance, estimated peak times from the expression profiles fit by our model have been compared with the peaks times defined by the CycleBase database. The root mean square error (RMSE) between the estimated peaks and the CycleBase defined peaks is similar to DeLorean model (∼14.5).

3 Recovering Diffusion Pseudotime (DPT)

We have also run the GrandPrix model on Klein *et al.* (2015)'s single-cell RNA-seq data without an informative prior. The estimated pseudotime has a high rank correlation with both the actual capture time (0.92) and the diffusion pseudotime (0.94). Although the rank correlation between estimated pseudotime and diffusion pseudotime have similar values for both non-informative (Figure 4(**b**)) and informative priors (main paper, Fig.3(**b**)), the model with capture time as informative prior shows a closer to linear correspondence to DPT. This is reflected in a higher linear correlation for the informative prior model (0.964) compared to the non-informative prior model (0.931).

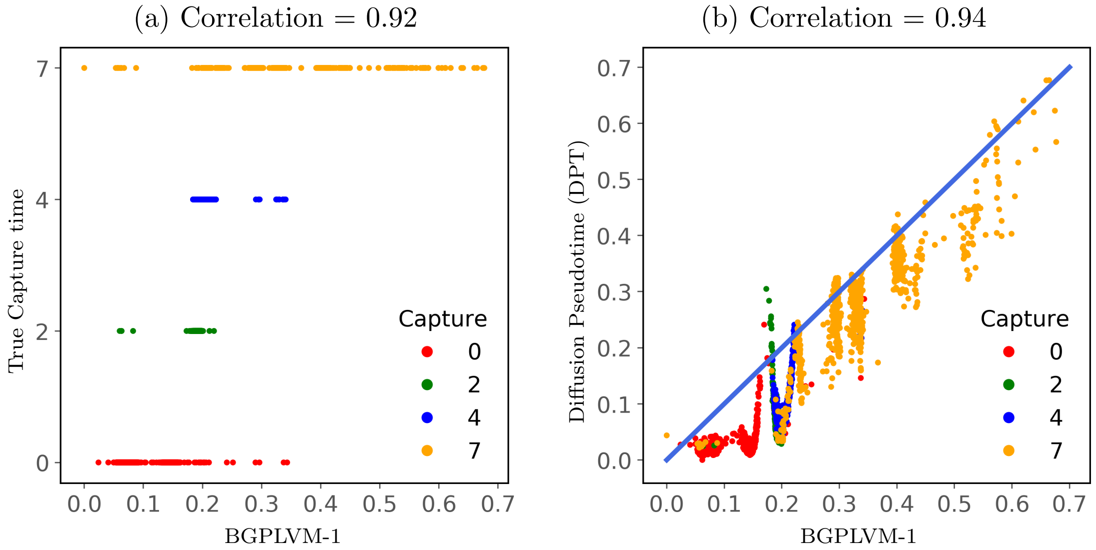

Figure 4: Mouse embryonic stem cells (Klein *et al.*, 2015): The model has been applied without using informative prior. The estimated pseudotime has very high correlations with the actual capture time as well as DPT, though the estimated pseudotime does not show similar density to DPT.

4 2-D visualization of ∼68k Peripheral Blood Mononuclear Cells (PBMCs)

Zheng *et al.* (2017) demonstrated the ability of single-cell transcriptomics to characterise large immune cell populations. They analysed the cellular heterogeneity present in ∼68k Peripheral Blood Mononuclear Cells (PBMCs). They used the top 1000 differentially expressed genes ranked by their normalised dispersion. First they used PCA to identify the first 50 principal components, then applied the k-means clustering algorithm and identified 10 distinct cell or cluster labels. They used t-SNE to visualise these 10 clusters in a two-dimensional projection. We have used these 2-D latent spaces from t-SNE to initialise our model. We have used 60 inducing points initialised via Latin Hypercube Sampling (LHS) (McKay *et al.*, 1979; Larson *et al.*, 2005). LHS is a statistical method that gives a near random sample from a multi-dimensional distribution. Figure 5 shows the 2-D BGPLVM visualisation of ∼68k PBMCc. Each cluster label represents the corresponding cluster from Zheng *et al.* (2017). Figure 5 (a) shows the estimated latent dimensions from the model that optimises the inducing points where as Figure 5 (b) corresponds to the model with fixed inducing points.

To highlight the importance of sensible initialisation we have also used PCA to initialise the model. Figure 6 shows a comparison of the ARI values between the predicted clustering from 2-D BGPLVM model (initialised using both PCA and t-SNE) and the true clustering for different number of inducing points used. In all the cases we have used the same experimental setup as well as same initialisation of the inducing variables. The model initialised using the t-SNE consistently possesses better ARI values than the model initialised with the PCA. Thus initialisation using the t-SNE is helping the model to discover better local optima.

The performance of VFE approximation is expected to improve with an increasing number of inducing points. Figure 6 shows that as we use more inducing points, we get a bigger ARI between the GrandPrix latent space clustering and the true clustering. However, using more inducing points requires extra memory and increases the number of model parameters. In Figure 6, we observe similar ARI values when optimising or fixing the inducing point locations given a sufficient number of inducing points (≥ 40). Thus when using more inducing points, optimising them does not significantly improve the model performance. Table 2 shows the number of iterations required by the model to converge and Table 3 shows the time required for each iteration for different number of inducing points. Using more inducing points improves the performance and fixing them reduces the computational cost per iteration without significantly affecting the number of iterations to convergence given enough inducing points (≥ 40). Conversely using a small number of inducing points fails to represent the entire complexity of

the system and it is beneficial to optimise their location.

Therefore, while working with larger datasets, the model must be initialised sensibly and inducing point location may be fixed when a sufficient number is used. Optimising inducing points jointly with model parameters and hyperparameters may find better minima but constraints on the available computational resources may make this impractical. Thus rather than optimising a small number of inducing points, using more carefully initialised but fixed inducing points may exhibit better performance. The inducing points can be chosen from a random subset of the model parameters or from a random multidimensional distribution. Additionally, if prior knowledge of the system is available, the number of inducing points may be set sensibly taking into account problem complexity such as discontinuities, changepoints, etc.

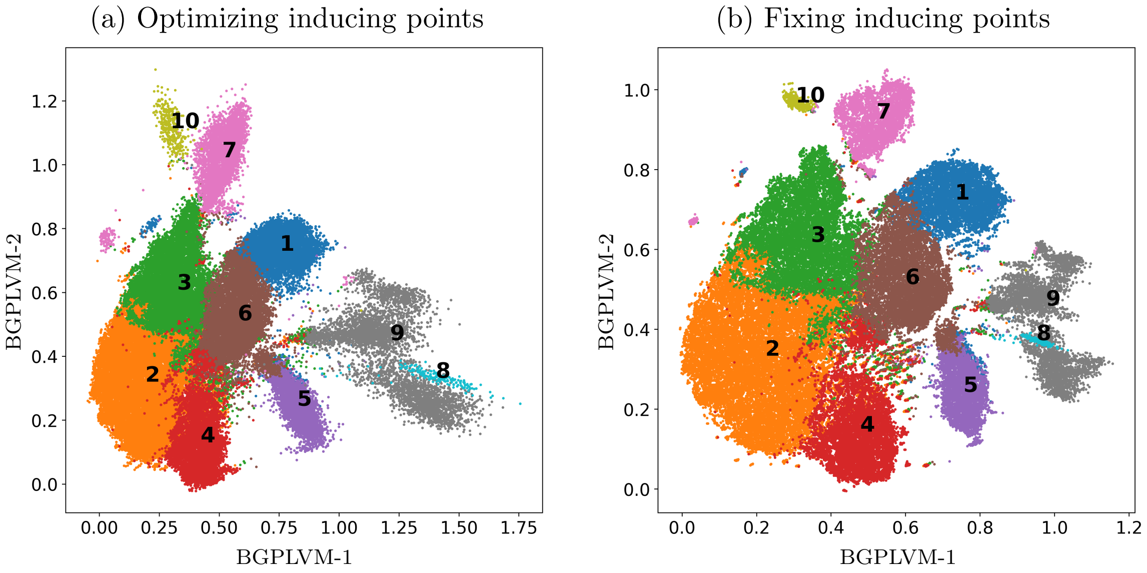

Figure 5: PBMCs with ∼68k cells (Zheng *et al.*, 2017): 2-D visualisation, the model is initialised using lower dimensional representation from t-SNE and uses 60 inducing points. (a): The model optimising the inducing points, has an ARI 0.538. (b): The model without optimising the inducing points, has an ARI 0.532. Both models have similar level of ARI values, while the latter converges faster. The cell clusters from Zheng *et al.* (2017) are also shown.

Table 2: Number of iterations to convergence required by the model initialised using t-SNE to optimise 2-D latent spaces from ∼68k PBMCs for different number of inducing points. The number of iterations reported are for both the models optimising and fixing the inducing points.

# inducing points	20	40	60
# iterations to converge: Optimizing inducing points	16	29	24
# iterations to converge: Fixing inducing points	56	27	28

Table 3: Fitting time required per iteration by the model initialised using t-SNE to optimise 2-D latent spaces from ∼68k PBMCs for different number of inducing points.

Number of inducing points	20	40	60
Approx. time (s)/iter: Optimizing inducing points	4.2	10.1	24.0
Approx. time (s)/iter: Fixing inducing points	2.7	9.3	19.1

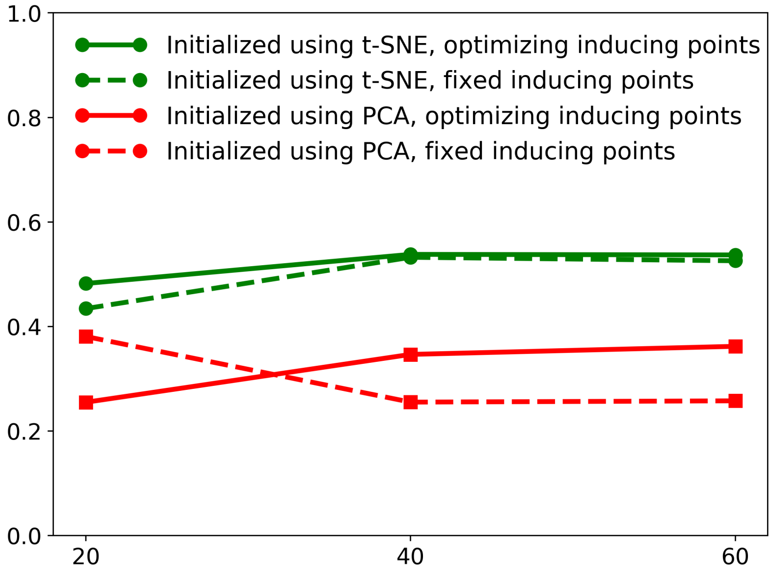

Figure 6: PBMCs with ∼68k cells (Zheng *et al.*, 2017): Comparison of the ARI values of the models initialised using the lower dimensional representation from both t-SNE and PCA for different number of inducing points. The performance has been analysed by optimising as well as fixing the inducing points.

5 Identifying branching dynamics in mouse embryonic stem cells

Guo *et al.* (2010) measure 437 differentiating cells across three distinct cell types TE, EPI and PE. We use the estimated pseudotime to visualise changes in the gene expression levels while the cells are progressing through differentiation stages. Zwiessele and Lawrence (2016) have listed genes that were differentially expressed for each pair of cell states, i.e. TE and EPI; PE and EPI; TE and PE. They listed the top 10 differentially expressed genes for each cell type transition. The marker genes of each cell type transition identified by Zwiessele and Lawrence (2016) are shown in Figures 7, 8 and 9. We plot the top 10 differentially expressed genes against the estimated pseudotime from the 2-D model for each combination of cell type transitions. Figure 10 is a heatmap which shows the expression profiles of all 48 genes across the estimated pseudotime and the second latent dimension. The heatmap also shows the gene clustering across cell capture stages. The differentially expressed genes among three cell states TE, EPI and PE can easily be identified in the heatmap.

We also investigate the robustness of the model with regards to the prior variance on pseudotime. In Figure 11, we show the accuracy and stability of the estimated pseudotime across a large range of prior variances. We also compare to the case when no prior is used. We use two measures to assess model robustness: rank correlation to the true experimental capture times (Figure 11(**a**)) and rank correlation to the pseudotime reported in the paper using prior variance 0.1. In both figures, the vertical black dashed line corresponds to the pseudotime at prior variance 0.1 which we have reported in the paper and the vertical red dashed line represents the model without a prior. The model is sufficiently robust across the range of prior variances considered. As the prior variance is increased, model performance gracefully deteriorates approaching the performance achieved by the no-prior model.

References

Bauer, M., van der Wilk, M., and Rasmussen, C. E. (2016). Understanding probabilistic sparse gaussian process approximations. In *Advances in Neural Information Processing Systems*, pages 1533–1541.

Blei, D. M., Jordan, M. I., *et al.* (2006). Variational inference for dirichlet process mixtures. *Bayesian analysis*, **1**(1), 121–143.

Blei, D. M., Kucukelbir, A., and McAuliffe, J. D. (2017). Variational inference: A review for statisticians. *Journal of the American Statistical Association*, **112**(518), 859–877.

Braun, M. and McAuliffe, J. (2010). Variational inference for large-scale models of discrete choice. *Journal of the American Statistical Association*, **105**(489), 324–335.

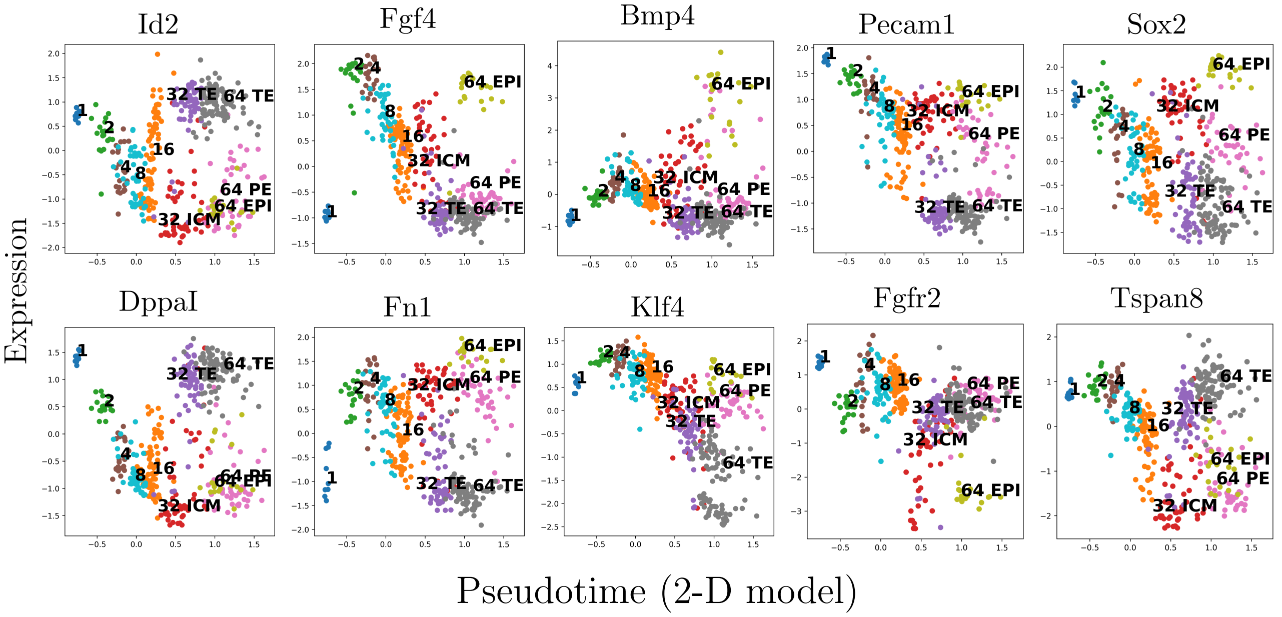

Figure 7: Single-cell qPCR of early developmental stages (Guo *et al.*, 2010): Top 10 differentially expressed genes between the stages TE and EPI.

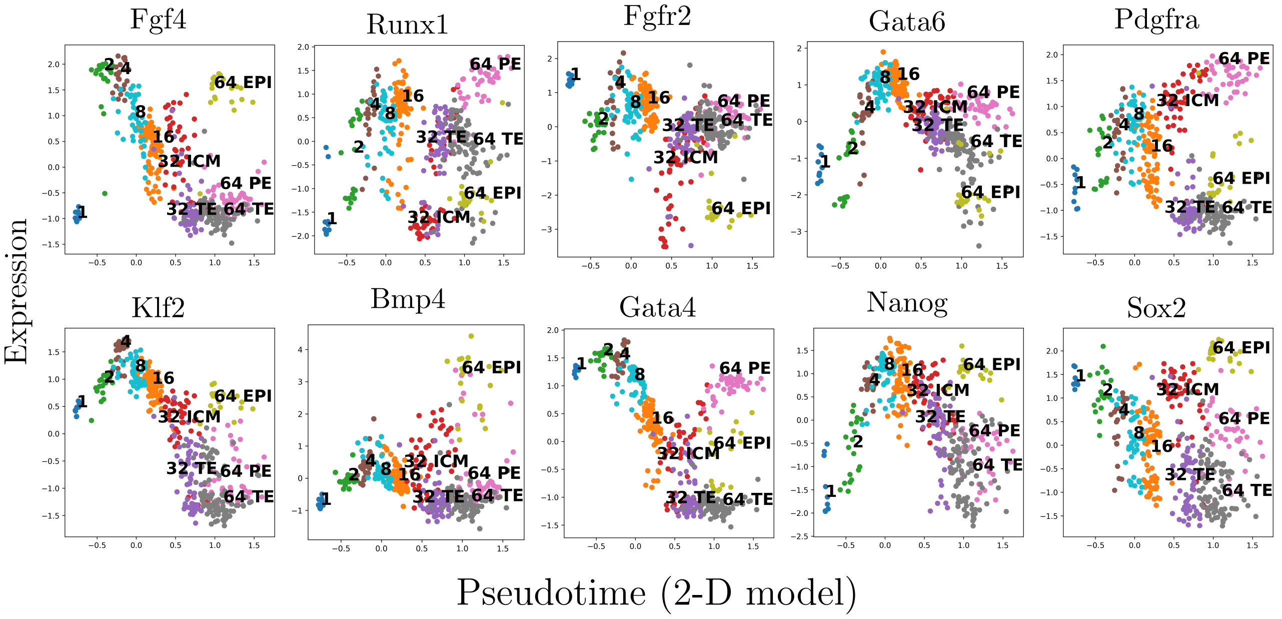

Figure 8: Single-cell qPCR of early developmental stages (Guo *et al.*, 2010): Top 10 differentially expressed genes between the stages PE and EPI.

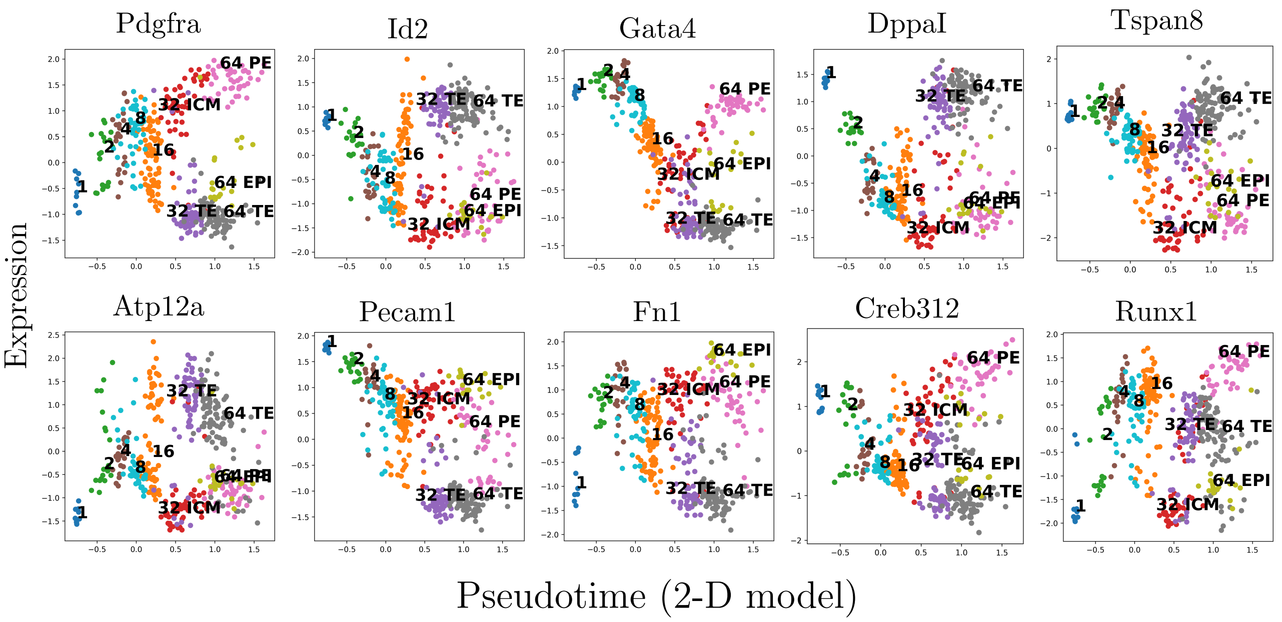

Figure 9: Single-cell qPCR of early developmental stages (Guo *et al.*, 2010): Top 10 differentially expressed genes between the stages TE and PE.

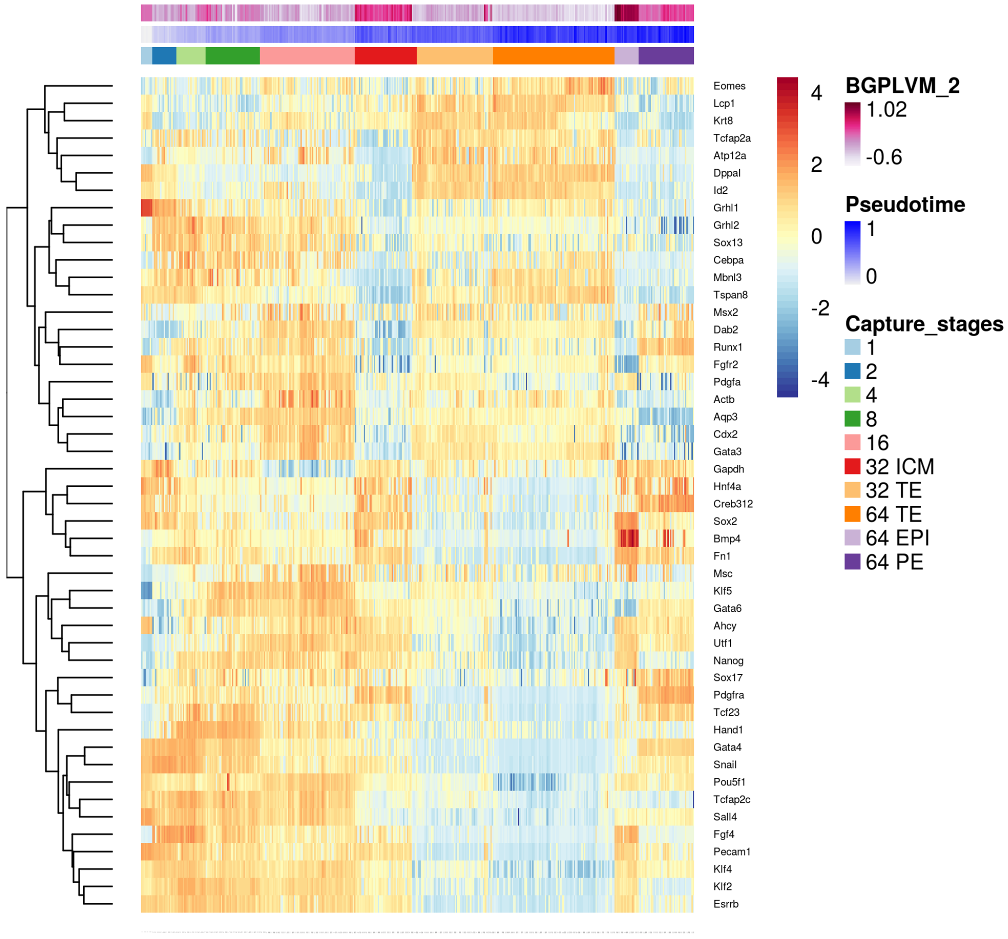

Figure 10: Single-cell qPCR of early developmental stages (Guo *et al.*, 2010): Heatmap showing the expression profiles of 48 genes across the pseudotime as well as added extra latent dimension.

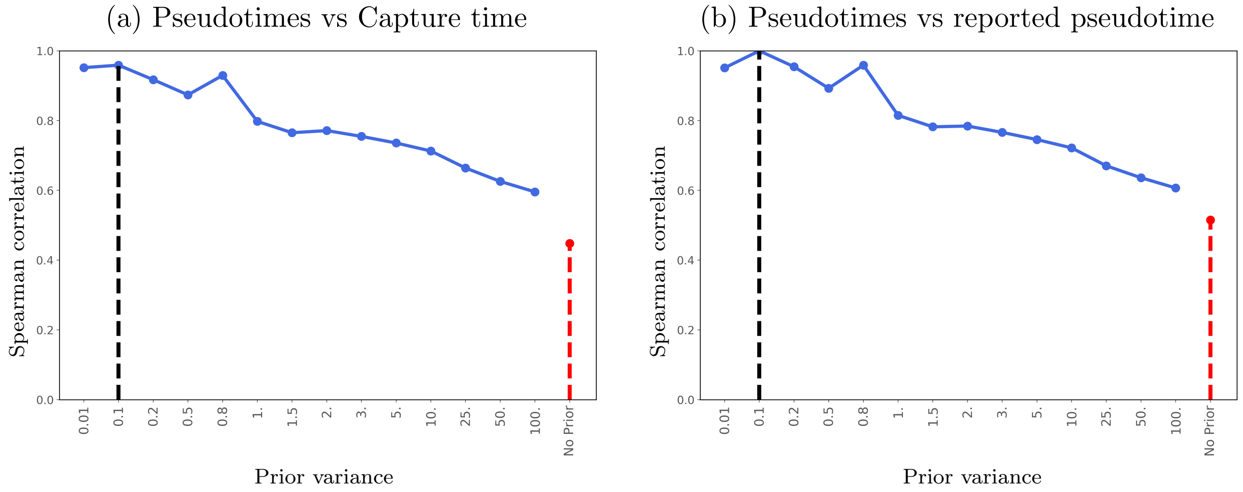

Figure 11: Single-cell qPCR of early developmental stages (Guo *et al.*, 2010): Effect of changing the prior variance (horizontal axis) on the estimated pseudotime. The vertical black dashed line represents the prior variance value (0.1) used in the paper and the vertical red dashed lines represent the no prior model. (a): Rank correlation (vertical axis) between the actual capture time and the estimated pseudotime. (b) Rank correlation (vertical axis) between the estimated pseudotime points and the pseudotime reported in the paper using prior variance = 0.1. The model with informative prior is more accurate even when using a large prior variance compared to the model without a prior.

Campbell, K. and Yau, C. (2016). Order under uncertainty: robust differential expression analysis using probabilistic models for pseudotime inference. *PLoS Computational Biology*, **12**(11).

Dai, Z., Damianou, A., Hensman, J., and Lawrence, N. (2014). Gaussian process models with parallelization and gpu acceleration. *arXiv preprint arXiv:1410.4984*.

Damianou, A. C., Titsias, M. K., and Lawrence, N. D. (2016). Variational inference for latent variables and uncertain inputs in gaussian processes. *The Journal of Machine Learning Research*, **17**(1), 1425–1486.

Duvenaud, D., Lloyd, J. R., Grosse, R., Tenenbaum, J. B., and Ghahramani, Z. (2013). Structure discovery in nonparametric regression through compositional kernel search. *arXiv preprint arXiv:1302.4922*.

Guo, G., Huss, M., Tong, G. Q., Wang, C., Sun, L. L., Clarke, N. D., and Robson, P. (2010). Resolution of cell fate decisions revealed by single-cell gene expression analysis from zygote to blastocyst. *Developmental cell*, **18**(4), 675–685.

Klein, A. M., Mazutis, L., Akartuna, I., Tallapragada, N., Veres, A., Li, V., Peshkin, L., Weitz, D. A., and Kirschner, M. W. (2015). Droplet barcoding for single-cell transcriptomics applied to embryonic stem cells. *Cell*, **161**(5), 1187–1201.

Kucukelbir, A., Tran, D., Ranganath, R., Gelman, A., and Blei, D. M. (2017). Automatic differentiation variational inference. *The Journal of Machine Learning Research*, **18**(1), 430–474.

Larson, V. E., Golaz, J.-C., Jiang, H., and Cotton, W. R. (2005). Supplying local microphysics parameterizations with information about subgrid variability: Latin hypercube sampling. *Journal of the atmospheric sciences*, **62**(11), 4010–4026.

Lawrence, N. (2005). Probabilistic non-linear principal component analysis with gaussian process latent variable models. *Journal of Machine Learning Research*, **6**(Nov), 1783–1816.

McDavid, A., Dennis, L., Danaher, P., Finak, G., Krouse, M., Wang, A., Webster, P., Beechem, J., and Gottardo, R. (2014). Modeling bi-modality improves characterization of cell cycle on gene expression in single cells. *PLoS Comput Biol*, **10**(7), e1003696.

McKay, M. D., Beckman, R. J., and Conover, W. J. (1979). Comparison of three methods for selecting values of input variables in the analysis of output from a computer code. *Technometrics*, **21**(2), 239–245.

Reid, J. E. and Wernisch, L. (2016). Pseudotime estimation: deconfounding single cell time series. *Bioinformatics*, **32**(19), 2973–2980.

Saelens, W., Cannoodt, R., Todorov, H., and Saeys, Y. (2018). A comparison of single-cell trajectory inference methods: towards more accurate and robust tools. *bioRxiv*, page 276907.

Santos, A., Wernersson, R., and Jensen, L. J. (2014). Cyclebase 3.0: a multi-organism database on cell-cycle regulation and phenotypes. *Nucleic acids research*, page gku1092.

Shalek, A. K., Satija, R., Shuga, J., Trombetta, J. J., Gennert, D., Lu, D., Chen, P., Gertner, R. S., Gaublomme, J. T., Yosef, N., *et al.* (2014). Single-cell rna-seq reveals dynamic paracrine control of cellular variation. *Nature*, **510**(7505), 363–369.

Snelson, E. and Ghahramani, Z. (2006). Sparse gaussian processes using pseudo-inputs. In *Advances in neural information processing systems*, pages 1257–1264.

Titsias, M. K. (2009). Variational learning of inducing variables in sparse gaussian processes. In *International Conference on Artificial Intelligence and Statistics*, pages 567–574.

Titsias, M. K. and Lawrence, N. D. (2010). Bayesian gaussian process latent variable model. In *International Conference on Artificial Intelligence and Statistics*, pages 844–851.

Windram, O., Madhou, P., McHattie, S., Hill, C., Hickman, R., Cooke, E., Jenkins, D. J., Penfold, C. A., Baxter, L., Breeze, E., *et al.* (2012). Arabidopsis defense against botrytis cinerea: chronology and regulation deciphered by high-resolution temporal transcriptomic analysis. *The Plant Cell*, **24**(9), 3530–3557.

Zheng, G. X., Terry, J. M., Belgrader, P., Ryvkin, P., Bent, Z. W., Wilson, R., Ziraldo, S. B., Wheeler, T. D., McDermott, G. P., Zhu, J., *et al.* (2017). Massively parallel digital transcriptional profiling of single cells. *Nature communications*, **8**, 14049.

Zwiessele, M. and Lawrence, N. D. (2016). Topslam: Waddington landscape recovery for single cell experiments. *bioRxiv*.
